# The Willingness toward Vaccination: A Focus on Non-Mandatory Vaccinations

**DOI:** 10.3390/vaccines11040828

**Published:** 2023-04-11

**Authors:** Angelo Capodici, Francesco Sanmarchi, Nicole Bonaccorso, Claudio Costantino, Elisa Maietti

**Affiliations:** 1 Department of Biomedical and Neuromotor Sciences, University of Bologna, 40126 Bologna, Italy; 2 Department of Health Promotion, Maternal and Infant Care, Internal Medicine and Medical Specialties “G. D’Alessandro”, Section of Hygiene, University of Palermo, Via Del Vespro 133, 90127 Palermo, Italy

**Keywords:** willingness, hesitancy, risk factors, COVID, society, herd immunity, vaccine effectiveness

## Abstract

The Special Issue “The Willingness toward Vaccination: A Focus on Non-mandatory Vaccinations”, published in the journal Vaccines, has the main aim of gathering more data on vaccine hesitancy and the willingness of individuals to receive vaccinations, particularly in the context of non-mandatory vaccines. The aim is to address vaccine hesitancy and improve vaccine coverage rates, in addition to identifying the determinants of vaccine hesitancy itself. This Special Issue garners articles that examine the external and internal factors that can influence the decision-making process of individuals regarding vaccination. Given that vaccine hesitancy is present in a significant part of the general population, it is crucial to have a better analytical understanding of the areas where hesitancy arises to determine appropriate strategies to address this issue.

## 1. Background

Vaccination is an indispensable public health tool, the significance of which cannot be overstated. The triumph of vaccines in the eradication or amelioration of devastating infectious diseases, such as smallpox, polio, and measles, is a testament to their remarkable efficacy [1]. However, vaccination acceptance, especially concerning non-mandatory vaccines, remains a vexing and pressing issue confronting contemporary society. Understanding vaccine hesitancy is imperative for formulating efficacious vaccination campaigns, achieving herd immunity, and safeguarding our communities.

The World Health Organization (WHO) identifies vaccine hesitancy as a growing concern, defined as the “delay in acceptance or refusal of vaccination despite the availability of vaccination services” [2]. Hesitancy arises from various sources, including misinformation, mistrust in healthcare professionals, religious convictions, and concerns about individual autonomy [3]. Tackling these multifarious challenges necessitates evidence-based strategies that operate at multiple levels, transfer best practices to every relevant context, and evaluate interventions within specific populations or settings to determine the efficacy of implementable actions that can be applied to the general population [4].

Education is a crucial aspect of promoting vaccination willingness. Evidence suggests that individuals with higher health literacy levels are more likely to accept vaccination [5]. Public health organizations and healthcare providers must collaborate to develop targeted educational campaigns that dispel popular misconceptions, emphasize vaccine benefits, and disseminate factual information on potential side effects [6].

Social norms and community engagement are also instrumental in fostering vaccine acceptance. Studies demonstrate that social influence and peer pressure significantly impact vaccination decisions [7]. Consequently, community leaders, such as religious and pop personalities, can play a pivotal role in endorsing vaccination programs and nurturing a culture of vaccination acceptance. Public health campaigns should leverage these potential allies to cultivate positive social norms regarding vaccination, especially non-mandatory vaccination.

Addressing the concerns of vaccine safety and efficacy is paramount. Building trust in regulatory agencies and the medical community is essential for fostering vaccination willingness. Transparent communication on vaccine development, approval processes, and post-licensure surveillance can alleviate concerns and establish trust [8].

## 2. Manuscripts Included in the Special Issue

At the end of March 2023, three manuscripts underwent a thorough peer review process, after which they were accepted for publication in the Special Issue (SI) entitled “The Willingness toward Vaccination: A Focus on Non-mandatory Vaccinations” of *Vaccines*. All three articles were cross-sectional studies.

Several topics were addressed in this Special Issue, and all manuscripts have been published and are available online through open access publishing. These studies are reported in Table 1 in chronological order of publication, with the following main features: first author, main topic, country, timeframe, methodology, and the main findings of the study.

In the study conducted by Zhao et al. [9], the authors analyzed the impact of digital media exposure on influenza vaccination willingness. They found that digital media exposure has a significant impact on the perceived benefits of vaccines, which in turn has a significant effect on vaccination intentions. They concluded that digital media can be an essential tool in promoting vaccination campaigns, but accountability and fact adherence must also be considered.

Sezerol et al. [10] perused COVID-19 vaccine booster acceptance in older citizens. The authors did not find any association between sociodemographic characteristics and booster dose acceptance. Educational level, previous vaccination history, and experiences of side effects were, on the other hand, associated with booster dose uptake.

Finally, Sun et al. [11] analyzed how vaccine hesitancy was expressed on the social media “Weibo” in China from 2020 to 2022. In their analysis, the authors revealed that constraints, confidence, and calculation were the main drivers of hesitancy as expressed on social media. Drawing from these findings, the authors concluded that the government and public health organizations need to provide convenient vaccination conditions, enhance public trust in vaccines, and reduce the cost of vaccination to promote vaccines.

## 3. Conclusions

Healthcare providers assume a crucial role in fostering vaccination willingness. Reports from scientific studies suggest that a strong recommendation from a healthcare provider is one of the most persuasive factors in a patient’s decision to receive a vaccine [12]. Thus, it is crucial for providers to possess a comprehensive understanding of the vaccines they endorse and to be capable of addressing common inquiries and concerns. By fostering trust in their patients, through a patient-centered approach and by building a solid relationship with them, providers can increase the likelihood of vaccine acceptance.

Vaccine hesitancy is a multifaceted issue that is influenced by a range of factors. Nevertheless, through education, community engagement, and the cultivation of trust in healthcare providers and regulatory agencies, it may be possible to overcome vaccine hesitancy and encourage a culture of vaccination acceptance. This approach could result in higher vaccination rates, improved herd immunity, and more robust and resilient communities.

## Figures and Tables

**Table 1:** Description of the manuscripts accepted for the Special Issue “The Willingness toward Vaccination: A Focus on Non-mandatory Vaccinations” in chronological order of publication.

First Author	Main Topic	Country	Timeframe	Methodology	Main Findings
Zhao Q. [9]	Health beliefs influencing influenza vaccination intentions	China	Not stated, as it reflects the included questionnaires.	Cross-sectional study	Digital media exposure influences influenza vaccination intention and may provide insights into vaccine promotion efforts in countries.
Sezerol M.A. [10]	COVID-19 vaccine booster dose acceptance among older adults	Turkey	April–May 2022	Cross-sectional study	Acceptance of vaccines was found to be strongly associated with education level, the experience of side effects, prior vaccination history, and attitudes toward other vaccines. Conversely, the main reasons for vaccine hesitancy were concerns regarding contraindications, vaccine safety, and limited mobility.
Sun Y. [11]	COVID-19 vaccine hesitancy	China	From 2020 to 2022	Cross-sectional study	The reasons for vaccine hesitancy include access to information and vaccination services, concerns about physical and allergic reactions, and international news. Additionally, factors such as constraints, lack of trust, and risk–benefit calculations are significant causes of vaccine hesitancy.

## Data Availability

For all inquiries please contact the corresponding author, all data is already available within the special issue.
